# Sub‐bundle based analysis reveals the role of human optic radiation in visual working memory

**DOI:** 10.1002/hbm.26800

**Published:** 2024-08-02

**Authors:** Yanming Wang, Huan Wang, Sheng Hu, Benedictor Alexander Nguchu, Du Zhang, Shishuo Chen, Yang Ji, Bensheng Qiu, Xiaoxiao Wang

**Affiliations:** ^1^ Medical Imaging Center, Department of Electronic Engineering and Information Science University of Science and Technology of China Hefei China; ^2^ State Key Laboratory of Brain and Cognitive Science, Institute of Biophysics Chinese Academy of Sciences Beijing China; ^3^ Institute of Artificial Intelligence Hefei Comprehensive National Science Center Hefei China

**Keywords:** blood oxygen level‐dependent (BOLD), optic radiation (OR), population receptive field (pRF), white matter, working memory

## Abstract

White matter (WM) functional activity has been reliably detected through functional magnetic resonance imaging (fMRI). Previous studies have primarily examined WM bundles as unified entities, thereby obscuring the functional heterogeneity inherent within these bundles. Here, for the first time, we investigate the function of sub‐bundles of a prototypical visual WM tract—the optic radiation (OR). We use the 7T retinotopy dataset from the Human Connectome Project (HCP) to reconstruct OR and further subdivide the OR into sub‐bundles based on the fiber's termination in the primary visual cortex (V1). The population receptive field (pRF) model is then applied to evaluate the retinotopic properties of these sub‐bundles, and the consistency of the pRF properties of sub‐bundles with those of V1 subfields is evaluated. Furthermore, we utilize the HCP working memory dataset to evaluate the activations of the foveal and peripheral OR sub‐bundles, along with LGN and V1 subfields, during 0‐back and 2‐back tasks. We then evaluate differences in 2bk‐0bk contrast between foveal and peripheral sub‐bundles (or subfields), and further examine potential relationships between 2bk‐0bk contrast and 2‐back task d‐prime. The results show that the pRF properties of OR sub‐bundles exhibit standard retinotopic properties and are typically similar to the properties of V1 subfields. Notably, activations during the 2‐back task consistently surpass those under the 0‐back task across foveal and peripheral OR sub‐bundles, as well as LGN and V1 subfields. The foveal V1 displays significantly higher 2bk‐0bk contrast than peripheral V1. The 2‐back task d‐prime shows strong correlations with 2bk‐0bk contrast for foveal and peripheral OR fibers. These findings demonstrate that the blood oxygen level‐dependent (BOLD) signals of OR sub‐bundles encode high‐fidelity visual information, underscoring the feasibility of assessing WM functional activity at the sub‐bundle level. Additionally, the study highlights the role of OR in the top‐down processes of visual working memory beyond the bottom‐up processes for visual information transmission. Conclusively, this study innovatively proposes a novel paradigm for analyzing WM fiber tracts at the individual sub‐bundle level and expands understanding of OR function.

## Graphical abstract

We explored the retinotopic properties and the top‐down information transmission characteristics of the optic radiation (OR) sub‐bundles. We revealed that the blood oxygen level‐dependent (BOLD) signals of OR sub‐bundles can encode high‐fidelity visual information, underscoring the feasibility of assessing white matter functional activity at the tract sub‐bundle level. Moreover, the OR engages top‐down visual working memory processes beyond the bottom‐up processes for visual information transmission.

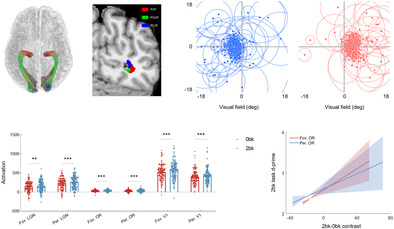

## 1 INTRODUCTION

White matter (WM), which consists of axons, carries information between gray matter (GM) and is crucial for brain function. Studying WM and their functional activities could help understand brain mechanisms of information processing. GM blood oxygen level‐dependent (BOLD) functional magnetic resonance imaging (fMRI) and its roles have widely been used in neuroscience research; however, research on WM BOLD signals and their significance is still in its infancy. Although the cerebral blood flow and volume in WM are relatively lower than in GM, the BOLD effect in WM is still detectable (Gawryluk et al., 2014; Gore et al., 2019). The temporal and spectral profiles and low‐frequency signal powers of WM BOLD signals in the resting state are comparable to those in the GM (Ding et al., 2013, 2016, 2018; Huang et al., 2020; Peer et al., 2017). Moreover, the BOLD effect of WM can be enhanced by tasks or stimulation and can be modulated by different stimulation parameters (stimulation intensity or frequency) (Ding et al., 2018; Huang et al., 2018; Li et al., 2020; Mishra et al., 2020; Wu et al., 2017). On this basis, it is now accepted that WM BOLD signals encode neural activity both during the resting state and under certain task or stimulation conditions. Now, the major challenge that needs to be addressed is that the majority of studies predominantly examined WM bundles as unified entities, thereby obscuring the functional heterogeneity inherent within these bundles.

Recently, we have discovered that two crucial visual fiber tracts, the optic radiation (OR) and the vertical occipital fasciculus (VOF), exhibit clear retinotopic maps at the voxel level (Wang et al., 2023), indicating that there are certain differences in the information transmitted within the WM fiber tracts. We postulate that there is functional heterogeneity inherent within WM fiber bundles. Hence, it is imperative to investigate the internal functional properties of fiber bundles. Previously, we explored the visual field maps of the OR and VOF using 178 subjects. We used the average BOLD signal of the group as the basis of WM BOLD signal since the typical BOLD signal of the WM has low signal‐to‐noise ratio (SNR). From this study and others, we learn that fitting the population receptive field (pRF) model to individual WM data is challenging, and thus, it is crucial to urgently develop innovative paradigms to explore the internal functional properties of WM fibers at the individual level.

Early studies in macaques and other species reported that the visual areas of both hemispheres are connected to corresponding topographic locations on the vertical meridian (Abel et al., 2000; Bosking et al., 2000; Olavarria, 1996). In addition, the topographical organization of the splenium of the corpus callosum originating from the left visual cortex closely corresponded to that originating from the right visual cortex (Dougherty et al., 2005; Saenz & Fine, 2010). Moreover, the connected lateral geniculate nucleus (LGN) and primary visual cortex (V1) neurons have similar receptive field characteristics (Sedigh‐Sarvestani et al., 2017). The topographic fidelity of connected regions suggests that fiber bundles themselves may also be topographically organized. Theoretically, axonal fibers are the carriers of neural activity, and their different locations carry the same functional information; thus, BOLD signals in long and continuous pathways should be temporally synchronized (Marussich et al., 2017). Moreover, studies based on spatio‐temporal functional correlation tensors have shown that the temporal correlation of BOLD signals in WM exhibits significant anisotropy (Ding et al., 2013, 2016). These studies suggest that the fiber bundle can be subdivided into sub‐bundles and that the SNR can be improved by averaging the BOLD signals within the sub‐bundles to explore the functional characteristics of the sub‐bundles at the individual level.

The OR plays a vital role in transmitting visual information and is one of the most important WM tracts in the visual system. We previously showed that the OR exhibits a clear functional topographic organization at the population level (Wang et al., 2023). Studies of autopsy‐based and diffusion magnetic resonance imaging (dMRI) fiber tracking studies have also revealed that the OR possesses a coarse structural topography (Ebeling & Reulen, 1988; Kammen et al., 2016). Therefore, this study combines dMRI and BOLD fMRI to evaluate the pRF characteristics of individual OR sub‐bundles and explore whether BOLD signals of individual‐level OR sub‐bundles can represent high‐fidelity visual information, thus investigating the feasibility of assessing WM function at the sub‐bundle level.

Of note, the differences between central and peripheral vision have extensively been investigated. For example, studies by Zhaoping (2017) and Bi et al. (2022) identified weaker top‐down feedback in the peripheral vision than in the central vision for visual information of recognition, color‐motion, and luminance‐motion. A top‐down process is also a critical cognitive component in working memory, characterized by manipulating sensory information momentarily to achieve the intended goal. Thus, whether there is a preference for central and peripheral visual fields in working memory processing requires further investigation. So far, studies have shown activation of V1 in visual working memory, suggesting the involvement of V1 in visual working memory (Lawrence et al., 2018). It is also important to note that the feedback layers of V1 target the LGN, which has recently been shown to process working memory information (Rahmati et al., 2023). Therefore, we hypothesize that the OR, which connects the V1 and LGN, is involved in the transmission of working memory information and that the central and peripheral parts of OR (i.e., separate OR sub‐bundles) encode distinct information associated with visual working memory. The detailed abbreviations and definitions used in the paper are listed in Table S1.

## 2 MATERIALS AND METHODS

### 2.1 Subjects

Based on the integrity of the fMRI and dMRI data in the 7T retinotopy dataset, 178 out of 181 subjects (109 women, 69 men) aged 22–35 years were selected for pRF analysis of the OR sub‐bundles. Eleven subjects were excluded due to the lack of the 3T HCP working memory dataset in the assessment of visual working memory. Each participant had normal or corrected‐to‐normal visual acuity.

### 2.2 HCP datasets

The 7T retinotopy dataset and 3T working memory dataset were downloaded from the HCP official website (https://db.humanconnectome.org). The datasets included: (1) Retinotopy Task fMRI Functional Preprocessed Extended, 7T BOLD‐fMRI data with 1.6 mm spatial resolution in standard MNI space; (2) Structural Preprocessed for 7T, same space with 7T BOLD‐fMRI data; (3) Diffusion Preprocessed, diffusion and structural data with 1.05 mm spatial resolution in the native acpc‐aligned space; (4) Structural Preprocessed, structural data with 0.7 mm spatial resolution preprocessed with the HCP structural pipeline in the native acpc‐aligned space; (5) Working Memory Task fMRI Preprocessed, 3T BOLD‐fMRI data with 2 mm spatial resolution in standard MNI space. For details of the HCP data acquisition and preprocessing, refer to the literature (Benson et al., 2018; Glasser et al., 2013; Van Essen et al., 2012, 2013; Vu et al., 2015). The ANTs (Advanced Normalization Tools) (Avants et al., 2011) were applied for image registration in this study.

### 2.3 Stimuli for the retinotopy dataset

The retinotopic mapping stimuli comprised a dynamic colorful texture (consisting of objects at multiple scales placed on a pink‐noise background) placed within slowly moving apertures, which can produce a high SNR in higher‐level visual areas. Apertures included clockwise or counterclockwise rotating wedges (RETCCW, RETCW), expanding or contracting rings (RETEXP, RETCON), or moving bars (RETBAR1, RETBAR2). The texture and aperture were constrained to a circular region with a diameter of 16.0° and were presented at a resolution of 768 × 768 pixels with a refresh rate of 15 Hz. The apertures were resized to 200 × 200 pixels to reduce the computational burden. For details, see Benson et al. (Benson et al., 2018).

### 2.4 Fiber tracking and segmentation

We reconstructed the left and right OR by using TractSeg that creates bundle‐specific tractograms by employing probabilistic tractography on tract‐specific fiber orientation distribution (FOD) function peaks (Wasserthal et al., 2018, 2019). This automated tracking technique was applied because it was trained on HCP data and can provide a balance between atlas‐based tracking and manual dissection approaches (Genc et al., 2020). The left and right OR were reconstructed with 25,000 streamlines per tract and then subdivided into 3 sub‐bundles based on the anatomical position of their termination in V1. Specifically, we used the approach proposed by Benson et al. to predict V1 and its pRF characteristics (eccentricity and polar angle) (Benson et al., 2014). The V1 was partitioned into 3 target subfields: foveal visual field (FVF, eccentricity ≤3°) subfield; peripheral upper visual field (PUVF, 3°< eccentricity ≤30° and 0°≤ polar angle <90°) subfield; peripheral lower visual field (PLVF, 3°< eccentricity ≤30° and 90°≤ polar angle <180°) subfield. The streamlines were associated with the subfields closest to their termination and a distance of less than 2 mm. The streamlines with a minimum distance between the termination and the subfields greater than 2 mm were not assigned to any sub‐bundles. Since the visual stimulus size of the retinotopy dataset used in this study was 8°, the far peripheral visual field could not be well evaluated. Considering the accuracy of model fitting and the integrity of the peripheral sub‐bundles, only V1 with eccentricity less than or equal to 30° was partitioned into subfields.

### 2.5 Masks for OR sub‐bundles

**FIGURE 1:**
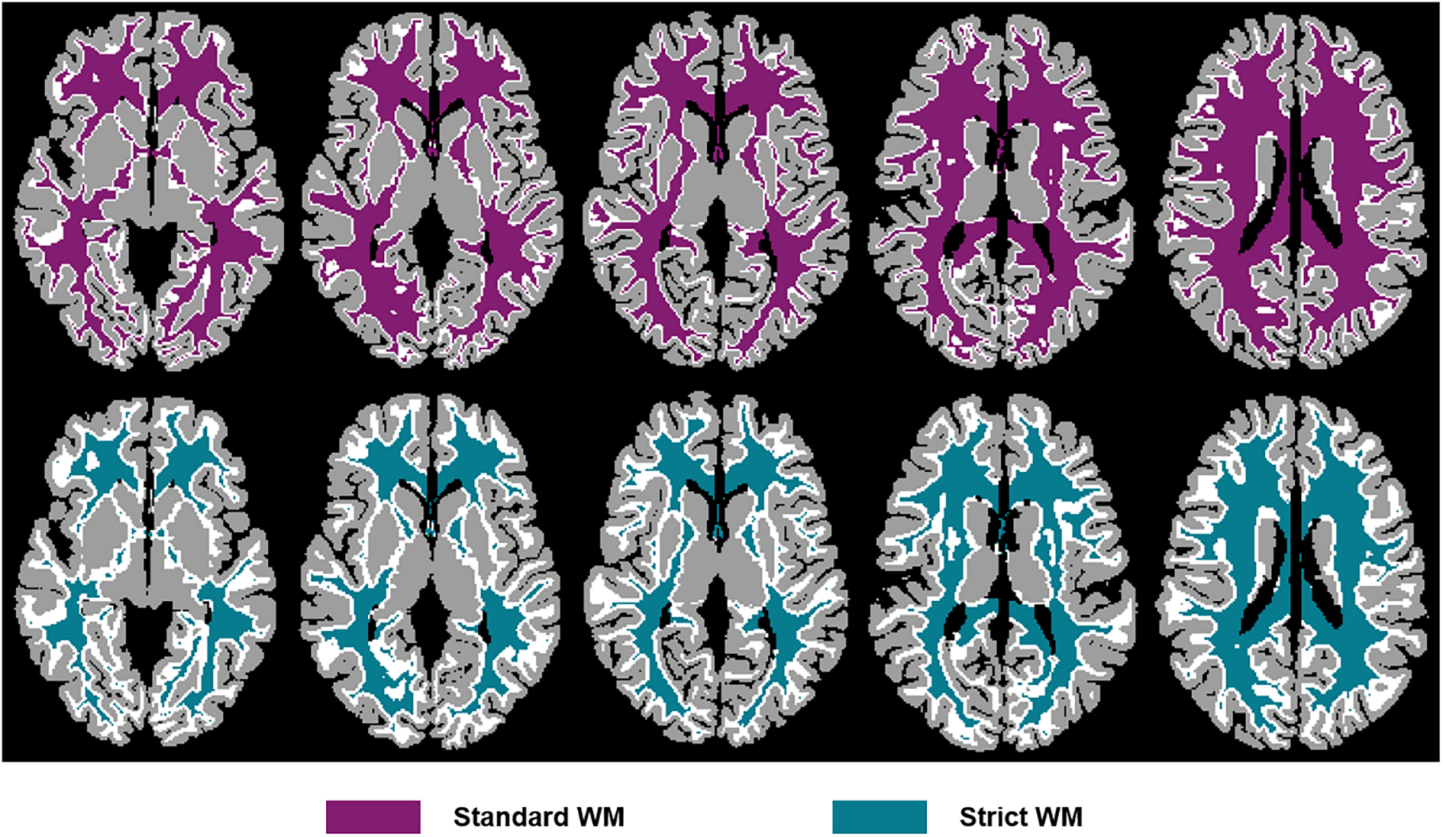
Axial view of standard and strict white matter (WM) masks. The gray matter (GM) mask is successively dilated two times, and the WM mask removes the overlapping voxels with the GM masks (dilated 1 and 2 times) to obtain the standard and strict WM masks. The GM and WM are rendered using gray and white colors, respectively. The standard (magenta) and strict (cyan) WM masks are represented in the upper and lower panels, respectively.

Each sub‐bundle was converted into a voxel‐level binary mask. It is noteworthy that fiber tracking process was performed at the sub‐voxel level. Thus, when converting the sub‐bundle into a voxel‐level mask, there were overlapping voxels between different sub‐bundle binary masks. The overlapping voxels need to be reallocated. To this end, the tckmap of MRtrix3 was first applied to map each sub‐bundle into a voxel‐level track‐weighted image (the number of streamlines in each voxel), and the overlapping voxels were subsequently assigned to the sub‐bundle containing the largest number of streamlines. In addition, to avoid contamination of adjacent GM voxels, we created WM masks with different degrees of restraint. The GM and WM masks were extracted based on the atlas of the native acpc‐aligned space generated by FreeSurfer and resampled to 1.05 mm. Next, the GM mask was successively dilated two times (one voxel at a time) using the 3dcalc of AFNI, and the WM mask removed the voxels that intersected with the GM mask dilated 1 and 2 times to obtain two WM masks with different constraint degrees, called standard and strict WM masks, respectively (Figure 1). Standard and strict individual sub‐bundle masks could be obtained by multiplying the sub‐bundles and the standard and strict WM masks. To ensure a higher SNR in WM BOLD and improve the fitting accuracy of the pRF model, we only included subjects whose all sub‐bundle masks were greater than ten voxels.

### 2.6 pRF analysis

The BOLD time series data was averaged across the voxels within each sub‐bundle. A pRF model called the Compressive Spatial Summation (CSS) (Kay et al., 2013) was applied to analyze the mean time series of each sub‐bundle. This model was implemented in the MATLAB toolbox, which is available on the Open Science Framework website (https://osf.io/bw9ec/). The model predicted the fMRI time series as the sum of a baseline time series and a stimulus‐related time series. The stimulus‐related time course was obtained by computing the dot product between a 2D isotropic Gaussian and the stimulus apertures, applying a static power‐law nonlinearity, scaling the result by a gain factor, and then convolving the result with a hemodynamic response function (HRF). This can be expressed formally as:

$$r\left(t\right)=\left(g\times {\left(S\left(t\right)\cdot G\right)}^{n}\right)*h\left(t\right),$$

where $r\left(t\right)$ is the predicted stimulus‐related time series, g is a gain parameter, $S\left(t\right)$ is the stimulus aperture at time t, n is an exponent parameter (fixed at 0.05 in the present study), $h\left(t\right)$ is an HRF, and G is the 2‐D isotropic Gaussian:

$$G=\exp\left(-\frac{{\left(x-x_{0}\right)}^{2}+{\left(y-y_{0}\right)}^{2}}{2\sigma^{2}}\right),$$

where $\left(x,y\right)$ represent different positions in the visual field, $\left(x_{0},y_{0}\right)$ are parameters controlling the position of the Gaussian, and σ is a parameter controlling the standard deviation of the Gaussian. This time series characterized BOLD modulations driven by the stimulus. The baseline time series, characterizing the baseline BOLD signal level, was obtained by computing a weighted sum of low‐order polynomial terms.

Notably, accurate evaluation of pRF relies on precise assumptions about the shape of the HRF (Lerma‐Usabiaga et al., 2020). WM HRF has reduced magnitudes, delayed onsets, and prolonged initial dips compared with connected cortical HRF (Fraser et al., 2012; Li et al., 2019; Tae et al., 2014). The WM HRF used in this study is the average of our reported voxel‐level OR HRF (Wang et al., 2023), where the HRF was a double‐gamma function, which was defined as the difference of two gamma probability density functions and similar to that in SPM (https://www.fil.ion.ucl.ac.uk/spm/). Five parameters were used to characterize the HRF curve using the following equation:

$$h\left(t\right)=\frac{t^{a_{1}-1}b_{1}^{a_{1}}e^{-b_{1}t}}{\Gamma \left(a_{1}\right)}-\frac{t^{a_{2}-1}b_{2}^{a_{2}}e^{-b_{2}t}}{c\Gamma \left(a_{2}\right)},$$

where Γ is the gamma function, $a_{1},b_{1},a_{2},b_{2}$, and c determine the shape of HRF, $a_{1}$ is the time to response, $a_{2}$ is the time to undershoot, $b_{1}$ is the response dispersion, $b_{2}$ is the undershoot dispersion, and c is the response undershoot ratio.

To reduce inaccuracies and biases due to local minima, a grid fit was first performed in which 10,400 plausible pRF parameter combinations were generated, including 25 nonlinearly spaced eccentricities, 32 polar angles, and 13 pRF sizes (all of these parameters were predefined in the toolbox), yielded 25 × 32 × 13 = 10,400 parameter combinations. The best‐fit parameter combination was then identified based on a least‐squares method. This parameter combination was subsequently used as the initial seed in a nonlinear optimization procedure (MATLAB Optimization Toolbox, Levenberg–Marquardt algorithm). We fitted the model to the data from each sub‐bundle. For each subject, two separate model fits were performed: one fit used standard sub‐bundles, and the other fit used strict sub‐bundles. Four quantities of interest were derived for each fit: pRF eccentricity ($\sqrt{\left(x_{0}^{2}+y_{0}^{2}\right)}$), pRF polar angle ($\tan^{-1}\left(y_{0}/x_{0}\right)$), pRF size ($\sigma /\sqrt{n}$), and percentage of explained variance ($R^{2}$). The model was fitted in pixel units, and the model parameters were thus converted to degrees by multiplying by a scaling factor of 16°/200 pixels. The pRF model and fitting procedures were extensively described in (Benson et al., 2018). Notably, the polar angle range obtained by this model was [0°, 360°), where the right upper visual field was [0°, 90°), the right lower visual field was [270°, 360°), the left upper visual field was [90°, 180°), and the left lower visual field was [180°, 270°).

The pRF parameters were threshed based on the explained variance values. The threshold was calculated by fitting the distribution of explained variance values to a Gaussian Mixture Model with two Gaussians (Benson et al., 2018; van Es et al., 2019); sub‐bundles with explained variance lower than the threshold likely reflected noise and were abandoned.

### 2.7 Assessment of visual working memory

We applied the HCP working memory dataset to evaluate the activation of the foveal and peripheral OR, LGN, and V1 sub‐regions under the 0‐back and 2‐back tasks. The HCP working memory dataset was acquired in two runs with left‐to‐right or right‐to‐left phase encoding direction. Data from both phase encoding directions was used to evaluate activation. The data preprocessing, including bandpass filtering (0.01–0.1 Hz) and detrending, was performed using AFNI (https://afni.nimh.nih.gov/). The LGN, OR, and V1 were subdivided into foveal and peripheral sub‐regions. The subdivision paradigms for V1 and OR remained consistent with those described in the above section. Specifically, the subdivision of V1 was based on the pRF properties reported by Benson et al. (2014), while the subdivision of OR was determined by considering the anatomical position of its termination in V1. In addition, the LGN was subdivided based on the pRF properties assessed in our previous study (Wang et al., 2023). The foveal and peripheral OR, V1, and LGN sub‐regions with 1.05 mm resolution in native acpc‐aligned space were co‐registered to the structural image with 1.6 mm in standard MNI space by using ANTs.

General linear model (GLM) analysis was performed to evaluate the activation of these sub‐regions. Notably, considering the differences between WM and GM HRF, the OR sub‐bundles were evaluated using double‐gamma HRF, whose parameters were fitted based on the average of our reported voxel‐level OR HRF (Wang et al., 2023), while the V1 and LGN subfields were evaluated using canonical HRF. We conducted a censoring procedure to exclude the time points at which the Euclidean norm of the first difference in motion effects exceeded 0.3 mm, as well as the previous time point. To further improve the GLM fitting accuracy, six rigid body parameters and CSF signals were regressed out.

### 2.8 Statistical analysis

To validate the rationality of subdividing sub‐bundles based on the pRF characteristics of their termination in V1, we evaluated the consistency of pRF characteristics between the sub‐bundles and the corresponding V1 subfields. We assessed the distribution of foveal and peripheral sub‐bundles eccentricities. Moreover, we evaluated whether the polar angle of the sub‐bundles calculated by the pRF model corresponded to their visual field labels and calculated the confusion matrix of this model fitting. In addition, to confirm that the pRF model fitting was not contaminated by adjacent GM signals, we evaluated the correlation of the eccentricity and polar angle between the strict sub‐bundles and standard sub‐bundles. Pearson correlation analysis was performed to evaluate the correlation of eccentricity. Due to the periodicity of the polar angle, the polar angle correlation of the left and right hemisphere sub‐bundles was evaluated using the circ_corrcc (Berens, 2009) in the MATLAB CircStat tool package.

For activation analysis of the OR sub‐bundles and LGN and V1 subfields, the subjects whose 2‐back task performance was less than 70% accurate were excluded. A paired‐sample t‐test was used to evaluate differences in activation between the 0‐back and 2‐back stimuli in the OR, V1, and LGN sub‐regions. Moreover, the 2bk‐0bk contrast differences of foveal and peripheral sub‐regions were assessed. Additionally, the d‐prime index was used to evaluate the accuracy of the 2‐back task (Haatveit et al., 2010). This index measures an individual's ability to detect signals, specifically evaluating sensitivity or discriminability as derived from signal detection theory, and is not influenced by response biases. It quantifies the normalized distance between the signal and noise and noise alone. Recognized as the “best” measure of psychophysical performance due to its bias‐free nature, the d‐prime index is essentially a standardized score. It is calculated as the difference between the Gaussian standard scores for the hit rate and the false‐alarm rate using the formula: d‐prime index = *Z*_hit_ − *Z*_false alarm_, where *Z* represents the inverse of the standard normal cumulative distribution function, evaluated at hit rate or false‐alarm rate. A d‐prime value of 3 indicates nearly perfect performance, while a value of 0 signifies chance (guessing) performance. We investigated the relationship between the 2‐back task d‐prime index and the 2bk‐0bk contrast. All analyses were corrected for multiple comparisons using a false discovery rate (FDR) at *p* < .05.

## 3 RESULTS

### 3.1 Fiber bundle segmentation

**FIGURE 2:**
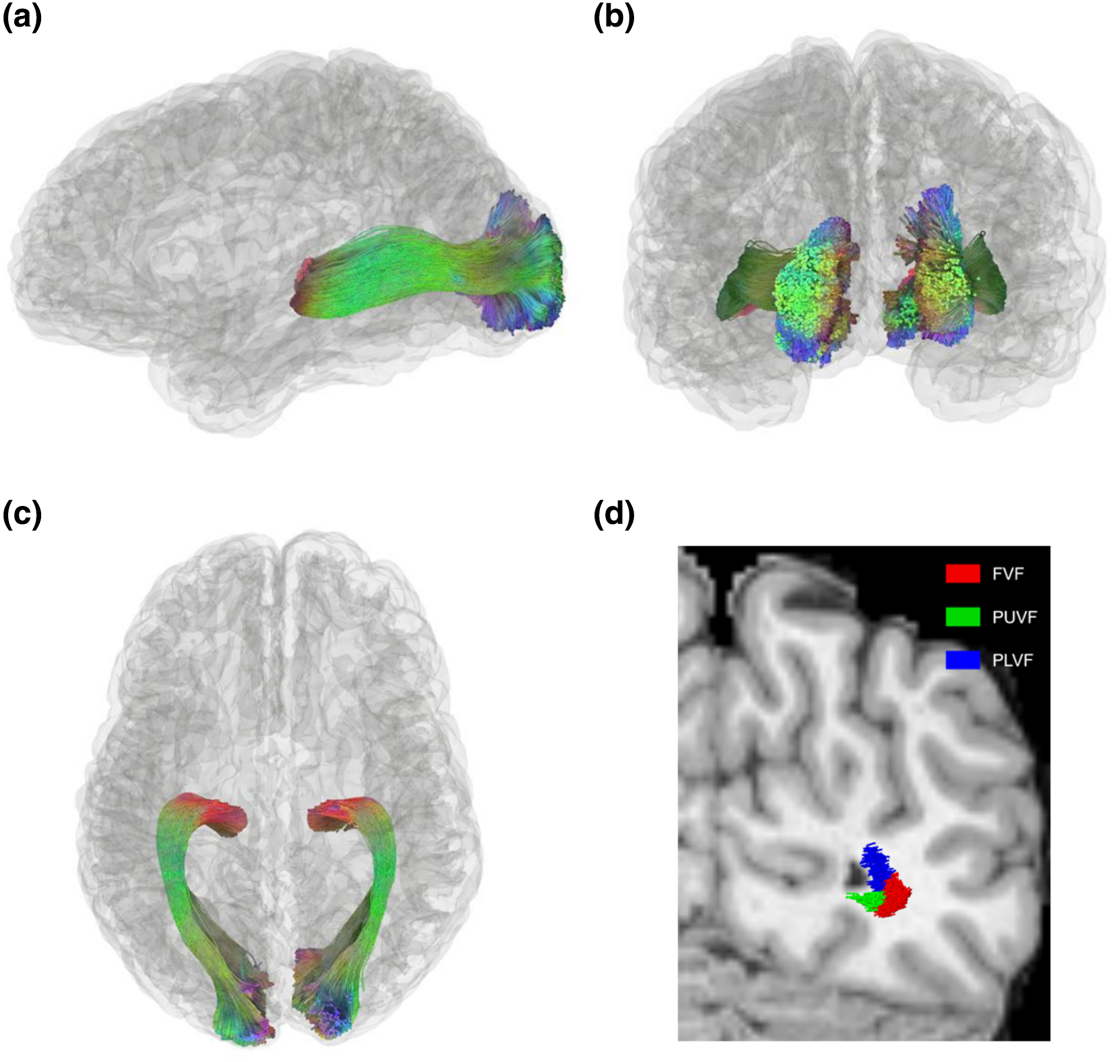
Reconstructed optic radiation (OR) and standard sub‐bundles. (a–c) Reconstructed OR in the sagittal, coronal, and axial planes. (d) Standard sub‐bundles on the T1 structural image: The foveal visual field (FVF) sub‐bundle (red), peripheral upper visual field (PUVF) sub‐bundle (green), and peripheral lower visual field (PLVF) sub‐bundle (blue). The foveal and peripheral visual field sub‐bundles are located on the lateral and medial sides of the brain, respectively, while the upper and lower visual field sub‐bundles are located on the inferior and superior sides of the brain, respectively.

The results illustrated that the OR can be accurately reconstructed by TractSeg (Figure 2a–c). A representative subject's standard sub‐bundles were rendered on the T1 structural image (Figure 2d): foveal visual field (FVF) sub‐bundle (red), peripheral upper visual field (PUVF) sub‐bundle (green), and peripheral lower visual field (PLVF) sub‐bundle (blue). Notably, the FVF sub‐bundle was located on the lateral side of the brain and in the middle portion of fiber bundle, while the peripheral visual field sub‐bundles (including the PUVF and PLVF sub‐bundles) were positioned on the medial side of the brain and outer portions of fiber bundle. Moreover, the upper visual field sub‐bundle and the lower visual field sub‐bundle were located on the inferior and superior sides of the brain, respectively. The relative position distribution of the sub‐bundles was consistent with previous post‐mortem dissection and dMRI studies (Ebeling & Reulen, 1988; Kammen et al., 2016).

**FIGURE 3:**
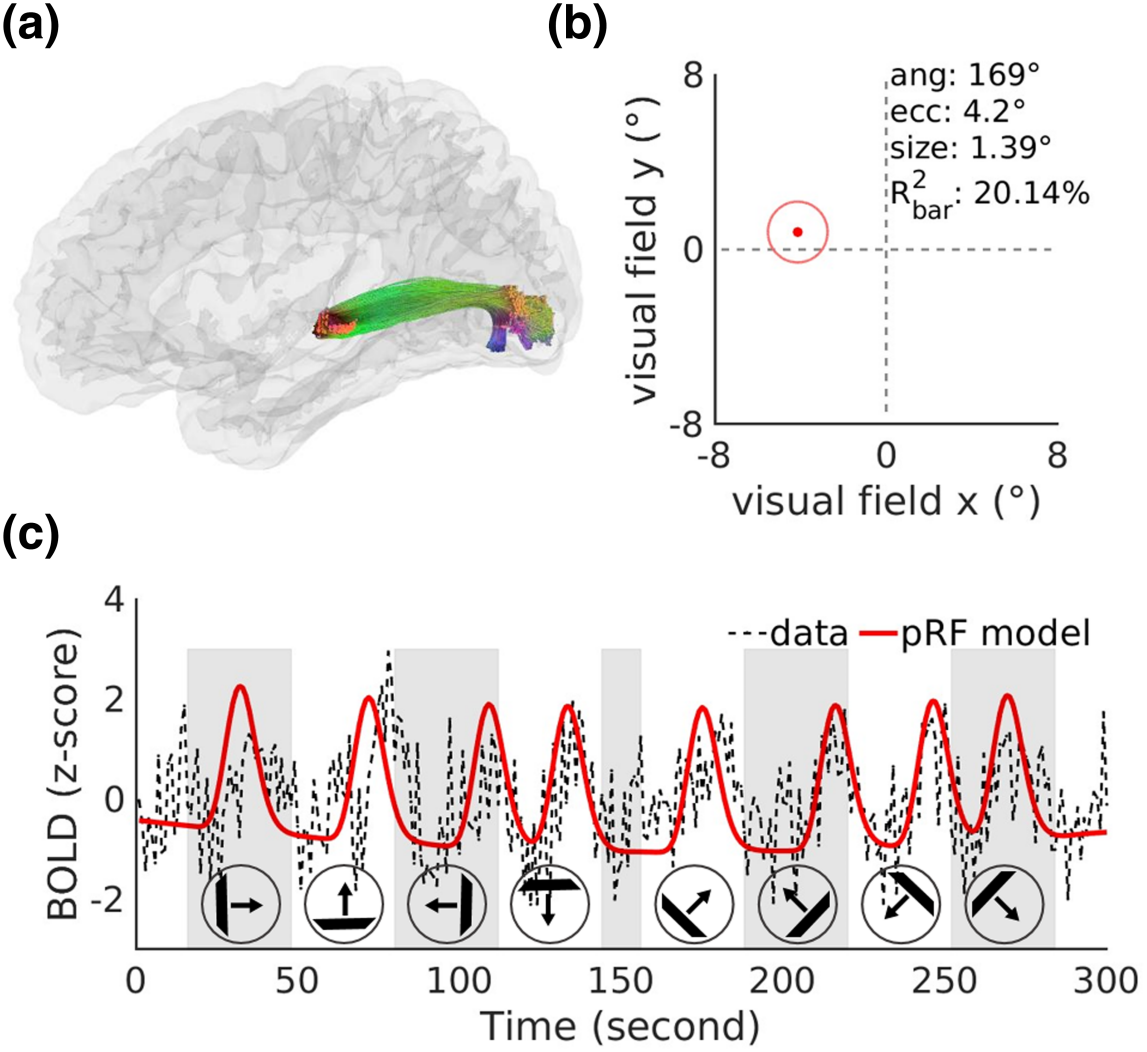
Population receptive field (pRF) fitting of a representative right peripheral upper visual field (PUVF) sub‐bundle. (a) The sagittal view of a representative sub‐bundle. (b) The pRF parameters of a representative sub‐bundle. The red dot and red circle indicate the position and size of the pRF, respectively. R (Gore et al., 2019) bar represents the explained variance between the original blood oxygen level‐dependent (BOLD) signal and the pRF model prediction. Good pRF model fitting is obtained for the BOLD signals of the representative sub‐bundle. (c) Coupling between the BOLD signal time series of a representative sub‐bundle (averaged BOLD signal time series across two runs of bar stimuli, black dashed line) and the pRF model prediction (red solid line).

In addition, the eccentricity and polar angle of a representative PUVF sub‐bundle from a subject's right hemisphere fitted by the pRF model were 4.2° and 169°, respectively (Figure 3a,b), matching the distribution of the labeled visual field (eccentricity ≥3°, 90°≤ polar angle ≤180°). Moreover, the explained variance of the model reached as high as 20.14%, indicating that BOLD signals in the visual WM sub‐bundles can be fitted reasonably well by the pRF model. The BOLD signal time series predicted by the pRF model was highly consistent with the original time series of the sub‐bundle (Figure 3c).

### 3.2 pRF properties of standard sub‐bundles

**FIGURE 4:**
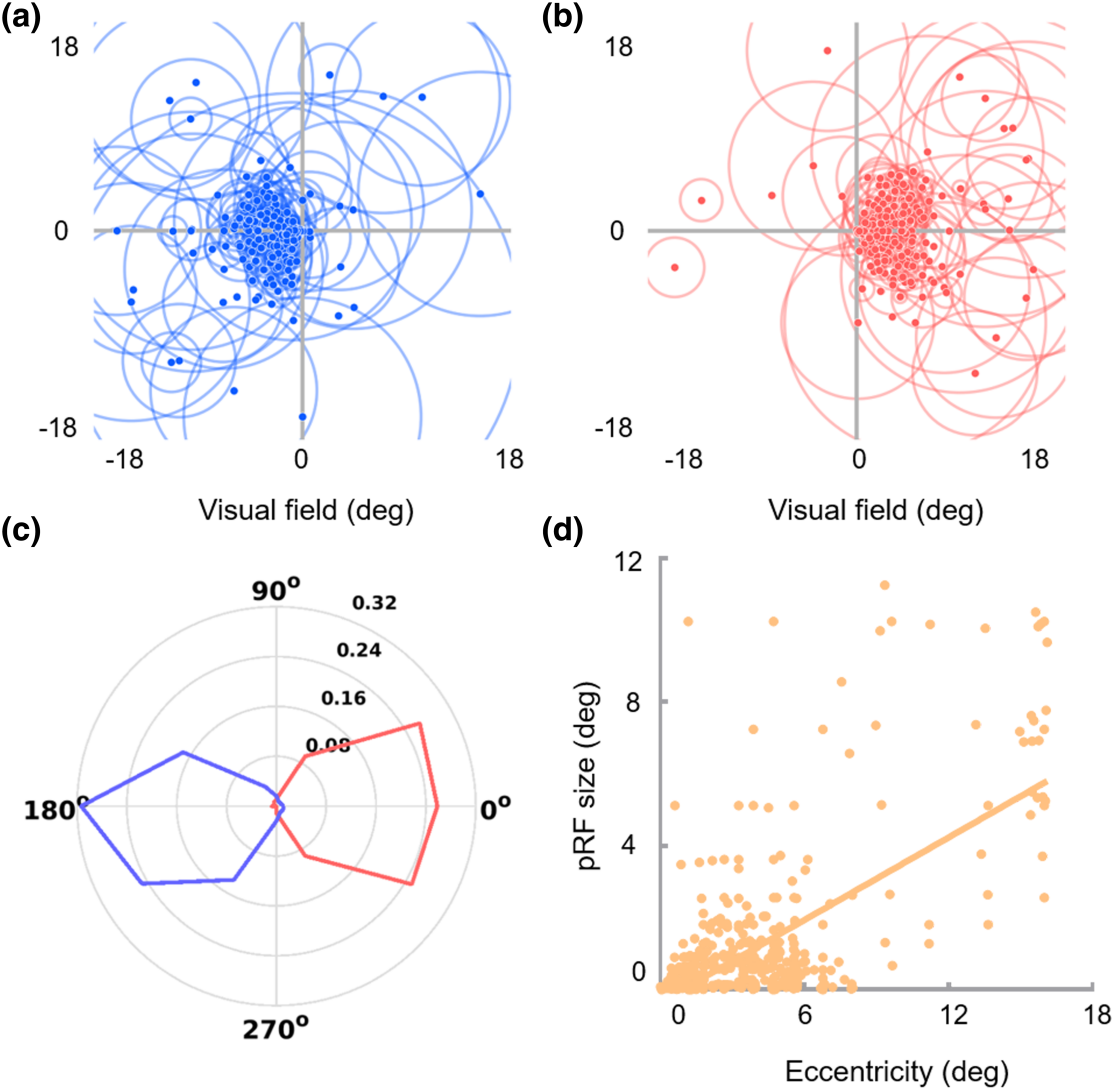
Distribution of population receptive field (pRF) properties in standard sub‐bundles. (a–c) Distribution of pRFs throughout the visual field. (a, b) Distribution of pRFs in the visual field for the right and left hemisphere sub‐bundles. Dots indicate the pRF centers; circles indicate the pRF size. (c) Distribution of polar angles. The solid lines represent the fractional volume of the polar angle (12 30° polar angle intervals, interval centers [0°, 30°, 60°, …, and 330°]), and the left hemisphere (red solid line) and right hemisphere (blue solid line) are plotted, respectively. (d) pRF eccentricity versus pRF size relations. Each dot represents a sub‐bundle. The pRF eccentricity is significantly positively correlated with the pRF size (*r* = .585, *p* < .001).

**FIGURE 5:**
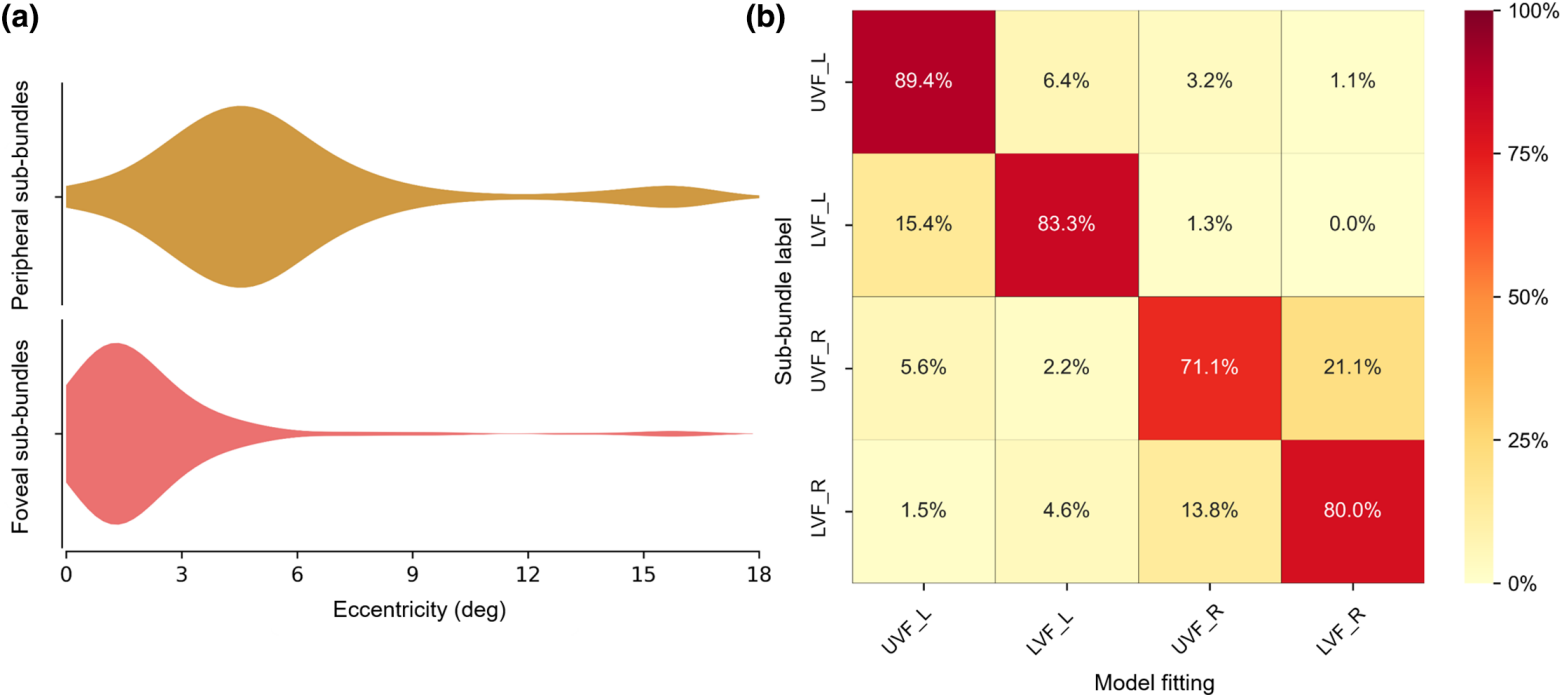
Distribution of population receptive field (pRF) eccentricities and confusion matrix of model fitting in standard sub‐bundles. (a) Distribution of pRF eccentricities. The eccentricities of the foveal sub‐bundles are mostly less than 3° (82.38%), and the eccentricities of the peripheral sub‐bundles are mostly greater than 3° (82.87%). (b) Confusion matrix of model fitting. The accuracy of the model fitting is 81.04%, which is much greater than the 4‐classification chance level accuracy (25%). Moreover, most of the incorrectly fitted polar angles are distributed in the adjacent visual fields. L, left; LVF, lower visual field; R, right; UVF, upper visual field.

The polar angles exhibited clear contralateral retinotopic organizations and were biased toward the horizontal meridian. The polar angles of the sub‐bundles of the right hemisphere were mostly distributed in the left visual field (93.57%, Figure 4a,c, blue), while the polar angles of the left hemisphere sub‐bundles were mostly distributed in the right visual field (97.79%, Figure 4b,c, red). Moreover, pRF eccentricity was significantly positively correlated with pRF size (*r* = .585, *p* < .001, Figure 4d). The representations of the contralateral visual field and the strong positive correlation between pRF eccentricity and pRF size were consistent with the standard retinotopic properties of OR (Wandell et al., 2007; Wang et al., 2023). Interestingly, the sub‐bundles and corresponding V1 subfields presented similar pRF properties. Specifically, the eccentricities of the foveal sub‐bundles were mainly distributed in the central visual field (eccentricity ≤3°, 82.38%), while the eccentricities of the peripheral sub‐bundles were mainly distributed in the peripheral visual field (eccentricity >3°, 82.87%) (Figure 5a). In addition, the model fitting accuracy of the polar angle of the sub‐bundles reached 81.04%, which was much higher than the 4‐classification chance level accuracy (25%), as shown in Figure 5b.

### 3.3 Relationships of the pRF properties between standard and strict sub‐bundles

**FIGURE 6:**
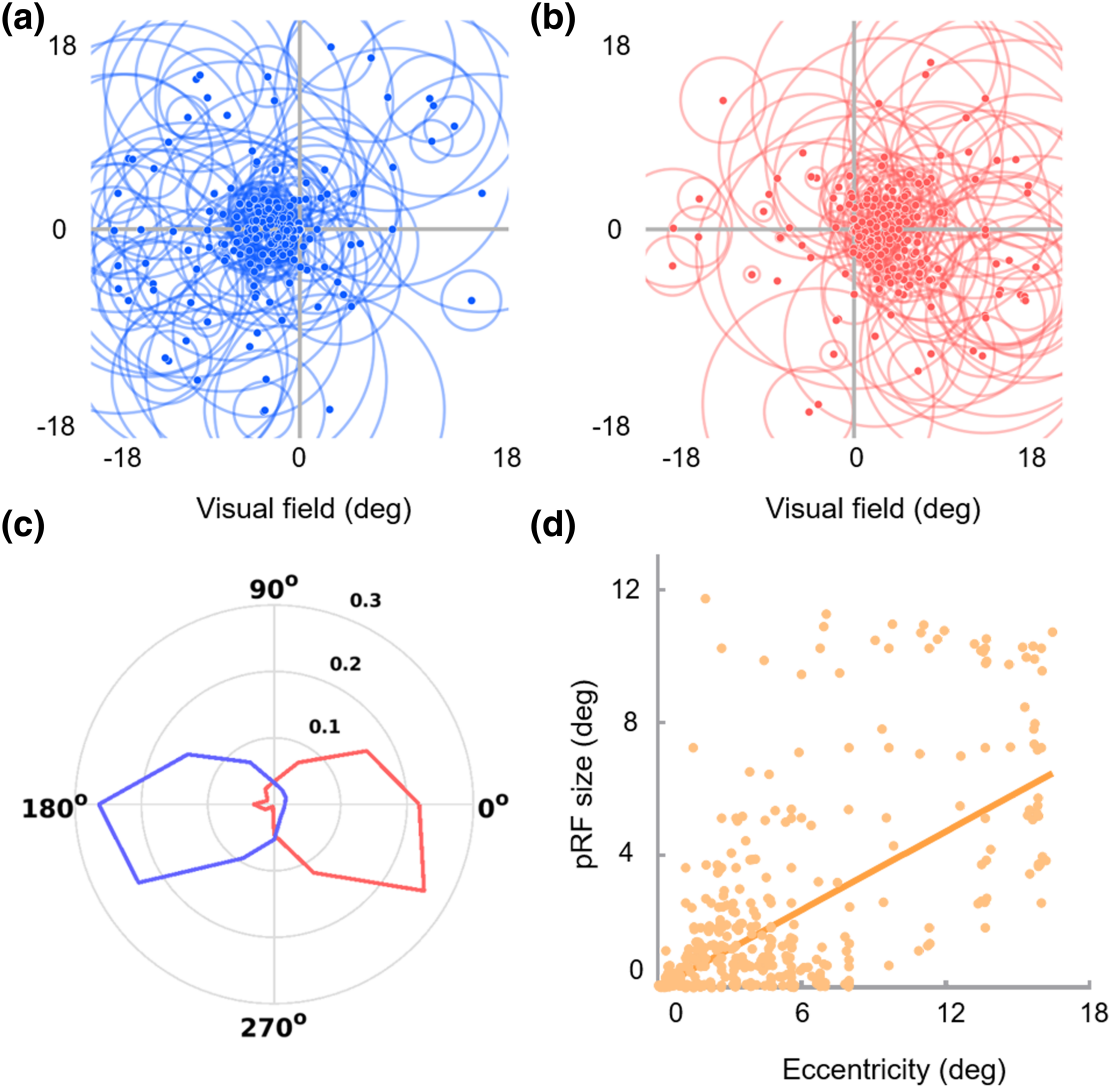
Distribution of population receptive field (pRF) properties in strict sub‐bundles. (a–c) Distribution of pRFs throughout the visual field. Dots indicate the pRF centers; circles indicate the pRF size. (a, b) Distribution of pRF in the visual field for the right and left hemisphere sub‐bundles. (c) Distribution of polar angles. The solid lines represent the fractional volume of the polar angle (12 30° polar angle intervals, interval centers [0°, 30°, 60°, …, and 330°]), and the left hemisphere (red solid line) and right hemisphere (blue solid line) are plotted, respectively. (d) pRF eccentricity versus pRF size relations. Each dot represents a sub‐bundle. The pRF eccentricity is significantly positively correlated with the pRF size (*r* = .622, *p* < .001).

To confirm that the pRF model fitting of sub‐bundles was not disturbed by adjacent GM signals, we also calculated the pRF parameters of strict sub‐bundles and evaluated their correlation with those of standard sub‐bundles. The results showed that the pRF properties of the strict sub‐bundles were also characterized by representations of the standard retinotopic properties of OR—the contralateral visual field representations—and increasing pRF size with eccentricity (*r* = .622, *p* < .001), as shown in Figure 6. Interestingly, the eccentricity (*r* = .733, *p* < .001) and polar angle (left hemisphere, *r* = .811, *p* < .001; right hemisphere, *r* = .825, *p* < .001) of strict sub‐bundles were significantly positively correlated with those of standard sub‐bundles.

### 3.4 Visual working memory activation analysis

**FIGURE 7:**
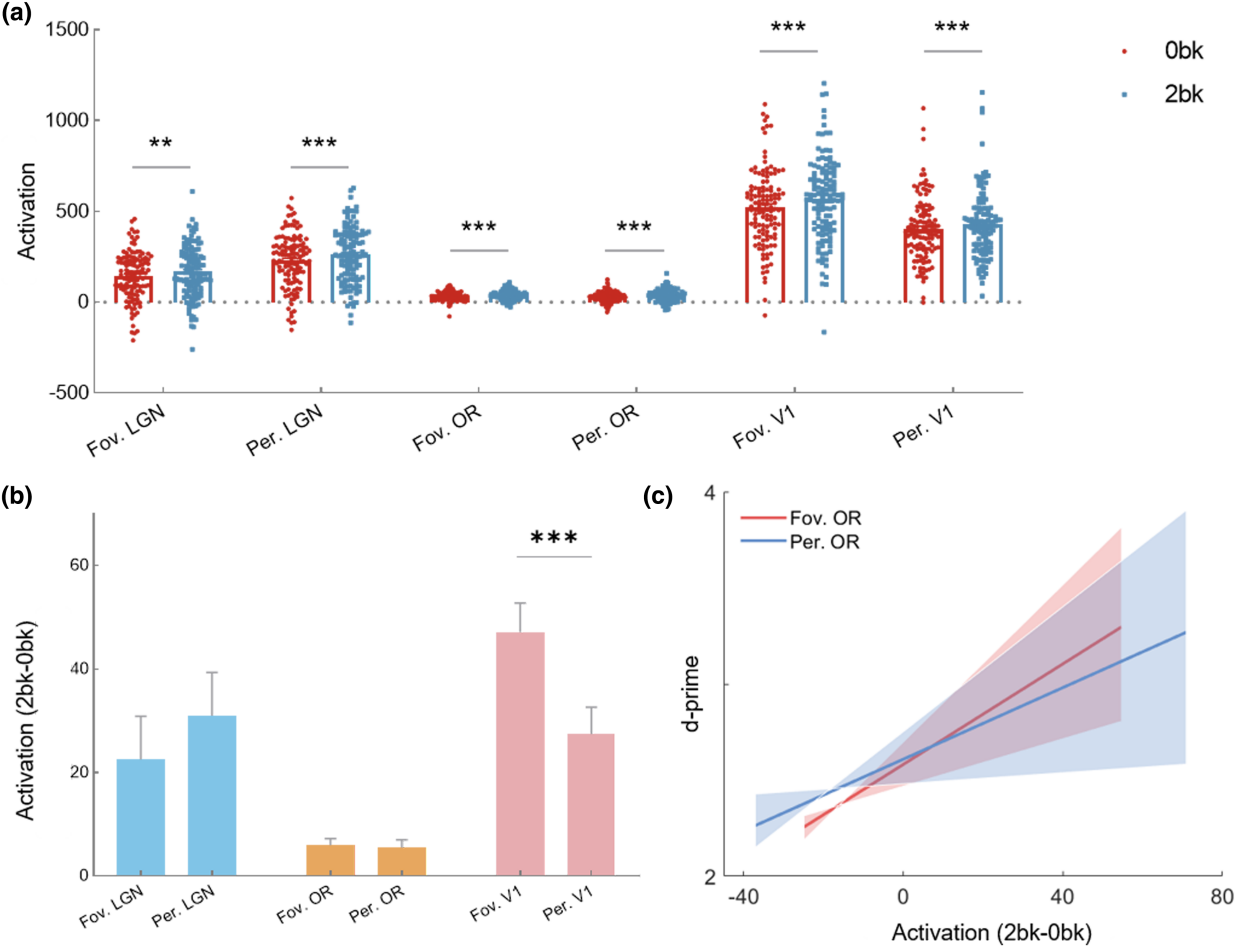
General linear model (GLM) activation analysis of foveal and peripheral optic radiation (OR) sub‐bundles and lateral geniculate nucleus (LGN) and V1 subfields in visual working memory. (a) 0‐back versus 2‐back activation differences in the OR sub‐bundles and LGN and V1 subfields. (b) Comparison of the 2bk‐0bk contrast between foveal and peripheral OR sub‐bundles and LGN and V1 subfields. The error bars depict ±1 SE from the mean. (c) Correlation between the 2‐back task d‐prime and the 2bk‐0bk contrast in foveal and peripheral OR sub‐bundles. Shaded areas represent bootstrapped 95% confidence intervals. Fov, foveal; Per, peripheral.

We compared the activation differences between 2‐back and 0‐back tasks and found that the activations under the 2‐back task were significantly stronger than those under the 0‐back task in foveal OR sub‐bundles (*p* < .001, *T* = 4.673), peripheral OR sub‐bundles (*p* < .001, *T* = 3.563), foveal LGN subfields (*p* = .008, *T* = 2.696), peripheral LGN subfields (*p* < .001, *T* = 3.733), foveal V1 subfields (*p* < .001, *T* = 8.416) and peripheral V1 subfields (*p* < .001, *T* = 5.287), as shown in Figure 7a. Given that the visual stimulus was almost the same under the 0‐back and 2‐back tasks, the activation induced by feedforward visual information input under these two tasks should be comparable. Thus, the 2bk‐0bk contrast mainly reflects the feedback signals from visual working memory. To assess the preference of visual working memory in foveal and peripheral sub‐regions, the 2bk‐0bk contrast differences between foveal and peripheral OR sub‐bundles and LGN and V1 subfields were evaluated. The findings demonstrated that the 2bk‐0bk contrast of foveal V1 subfields was significantly greater than that of peripheral V1 subfields (*p* < .001, *T* = 4.388) (Figure 7b). However, no differences were found in foveal and peripheral LGN or OR. Furthermore, we assessed whether the activation of visual working memory could serve as a predictor of performance in the 2‐back task. Interestingly, we found that the 2bk‐0bk contrast in only the foveal (*r* = .258, *p* = .023) and peripheral (*r* = .221, *p* = .041) OR sub‐bundles, not in the foveal and peripheral V1 and LGN subfields, was significantly positively correlated with the 2‐back task d‐prime (Figure 7c). The results illustrated that visual working memory activation, but mainly in the OR, holds promise as a predictor of performance in the 2‐back task.

## 4 DISCUSSION

Here we explore the retinotopic properties and the top‐down information transmission characteristics of OR sub‐bundles. The findings demonstrate that the distribution of pRF centers of OR sub‐bundles is characterized by representations of the contralateral visual field and that there is a strong positive correlation between pRF eccentricity and size. The pRF characteristics of both standard sub‐bundles and corresponding V1 subfields show a high degree of similarity. Further analyses reveal substantial correlations between the two pRF characteristics, that is, those of the standard sub‐bundles versus those of the strict sub‐bundles. These aforementioned findings suggest a robust representation of retinotopic properties by OR sub‐bundles. Furthermore, GLM analysis shows that the activation under the 2‐back task is more potent than that under the 0‐back task. The 2bk‐0bk contrast of foveal V1 is more intensified compared to that of peripheral V1. The 2bk‐0bk contrast in only the foveal OR and peripheral OR is significantly positively correlated with the 2‐back task d‐prime.

Our study adds the following innovative aspects to the study of the functional activity of WM. First, we propose using WM BOLD signals from sub‐bundles as an alternative to voxel‐level WM BOLD signals, which suffer from low SNR. We next add the concepts of polar angle and eccentricity to understand the pRF characteristics of OR sub‐bundles. The concept of polar angle and eccentricity in the subdivisions of OR is not entirely new. Previous studies of age‐related changes have applied a similar approach to subdivide OR into foveal, macular, and peripheral sub‐bundles to capture age‐related microstructural changes at the WM sub‐bundle level (Kruper et al., 2023). From the point of computation, the results of the subdivision of sub‐bundles in our study align with those presented in the previous studies of dMRI fiber tracking and autopsy (Ebeling & Reulen, 1988; Kammen et al., 2016). The eccentricity correlates with the pRF size, and the polar angle exhibits contralateral visual field representations, both demonstrating standard retinotopic properties (van Es et al., 2019; Wandell et al., 2007). Most misfitted polar angles fall within adjacent visual fields, and a strong bias of polar angles is seen around the horizontal meridian, similar to the previous observations of the studies of retinotopic properties based on voxel‐level analyses (Wang et al., 2023). The characteristics of V1 subfields have a significant level of similarity with eccentricity and polar angles of our OR sub‐bundles. These findings underscore the practicability of sub‐bundle subdivision based on the anatomical position of fiber tract termination in the cortex.

A major concern in WM BOLD studies is that activations are attributable to the confounding effects arising from adjacent GM and vasculature. To answer this question, we conducted our experiments with WM masks of different degrees of constraint to reduce the influence of adjacent GM structures. Our results show that WM activations are free from GM. In fact, the pRF properties of all sub‐bundles obtained at different degrees of constraints conform to the properties of standard pRF, and they have a high level of correlations. Taken together, these findings converge to confirm that the BOLD signals of the OR sub‐bundles can represent high‐fidelity visual information and that it is feasible to assess the functional properties of WM based on sub‐bundles. This study provides a paradigm for the analysis of the internal functional profiles of large WM fiber tracts (such as corticospinal tract and superior longitudinal fasciculus), that is, subdividing large WM fiber tracts into smaller sub‐bundles.

Of note is that the OR contains feedforward and feedback pathways (Briggs & Usrey, 2011). The feedforward pathway involves the transfer of visual signals from the retina to V1 via the LGN. In the feedback pathway, corticogeniculate neurones provide synaptic input to the LGN and the cortex to which the LGN projects. The physiological response characteristics of visual system neurons are inherited mainly from feedforward input. However, the number of feedback inputs exceeds the number of feedforward inputs, and the functional role of feedback inputs in visual information processing remains undetermined (Briggs, 2020). This study utilizes WM BOLD analysis to explore the function of foveal and peripheral OR sub‐bundles in visual working memory, an important feedback information processing function. The results suggest that for foveal and peripheral LGN, OR, and V1, 2‐back task activation is greater than 0‐back. These findings demonstrate that V1, LGN, and OR are indeed involved in visual working memory processes, supporting the sensory recruitment hypothesis (Zhao et al., 2022). A pronounced central preference in visual working memory is observed in V1, aligning with previous studies (Bi et al., 2022; Zhaoping, 2017). However, no distinct preference is found in the LGN and OR, indicating that central preference may solely exist in V1 and higher‐level brain areas, with no central preference in downstream areas of V1. More importantly, 2‐back task performance is associated with activation in the OR but not with activation in the LGN and V1. This implies that the OR may also play an important role in determining the quality of visual working memory. The reason behind this association may be attributed to the fact that the OR solely transmits information between the LGN and V1, resulting in cleaner signals compared to those of V1 and LGN.

This study has a few limitations worth noting. First, the lower resolution of dMRI images leads to voxel overlap between sub‐bundle masks. Utilizing higher‐resolution (submillimeter) images would be able to reduce voxel overlap and facilitate more precise segmentation of sub‐bundles. Second, it is still challenging for the existing dMRI fiber tracking paradigms to accurately track far peripheral V1‐connected OR, potentially impacting the analysis of peripheral sub‐bundles. Lastly, since the HCP working memory dataset does not include eye tracking data, the impact of eye movements on the analysis of visual working memory was not controlled.

## 5 CONCLUSION

In summary, this study explores the retinotopic properties and the top‐down information transmission characteristics of the OR sub‐bundles by using the pRF model and GLM analysis. The findings demonstrate that the BOLD signals of OR sub‐bundles encode high‐fidelity visual information, indicating the feasibility of assessing WM functional activity at the tract sub‐bundle level. Moreover, the OR not only transmits visual information from bottom to up but also engages top‐down cognitive processes, such as visual working memory.

## AUTHOR CONTRIBUTIONS

*The conception or design of the study*: Y.M.W., B.S.Q., and X.X.W. *Analysis or interpretation of data*: Y.M.W., H.W., S.H., D.Z., and S.S.C. *Drafting or revising of the paper*: Y.M.W., B.A.N., Y.J., and X.X.W. *Final approval of the version published*: B.S.Q. and X.X.W.

## CONFLICT OF INTEREST STATEMENT

None of the authors have a conflict of interest to disclose.

## Supporting information


**Data S1.** Supporting information.

## Data Availability

Data and codes are available upon request by contacting the lead contact, Xiaoxiao Wang (wang506@ustc.edu.cn).
