# Supplementary material for: Sub‐bundle based analysis reveals the role of human optic radiation in visual working memory
Source: Hum Brain Mapp. 2024 Aug 2;45(11):e26800. doi: 10.1002/hbm.26800 (PMC11295295; doi:10.1002/hbm.26800)
Supplement: Supplementary file 1 — Data S1. Supporting information. [file HBM-45-e26800-s001.docx]

**Table S1. List of abbreviations.**

| **Abbreviation** | **Definition** |
| --- | --- |
| ANTs | Advanced Normalization Tools |
| BOLD | Blood Oxygen Level-Dependent |
| CSS | Compressive Spatial Summation |
| dMRI | Diffusion Magnetic Resonance Imaging |
| FDR | False Discovery Rate |
| fMRI | Functional Magnetic Resonance Imaging |
| FOD | Fiber Orientation Distribution |
| Fov | Foveal |
| FVF | Foveal Visual Field |
| GLM | General Linear Model |
| GM | Gray Matter |
| HCP | Human Connectome Project |
| HRF | Hemodynamic Response Function |
| LGN | Lateral Geniculate Nucleus |
| OR | Optic Radiation |
| Per | Peripheral |
| PLVF | Peripheral Lower Visual Field |
| pRF | Population Receptive Field |
| PUVF | Peripheral Upper Visual Field |
| SNR | Signal-to-Noise Ratio |
| V1 | Primary Visual Cortex |
| VOF | Vertical Occipital Fasciculus |
| WM | White Matter |
